# Cysticercosis in free-ranging agoutis (*Dasyprocta leporina*) in the Eastern Brazilian Amazon

**DOI:** 10.1590/S1984-29612023044

**Published:** 2023-07-21

**Authors:** Alex Junior Souza de Souza, Andreza Pinheiro Malheiros, André Antônio Corrêa das Chagas, Max Moreira Alves, Marcella Katheryne Marques Bernal, Liliane Almeida Carneiro, Michele Soares Gomes-Gouvêa, Heloisa Marceliano Nunes

**Affiliations:** 1 Departamento de Patologia, Faculdade de Medicina Veterinária e Zootecnia, Universidade de São Paulo - USP, São Paulo, SP, Brasil; 2 Instituto de Ciências da Saúde, Universidade Paulista - UNIP, Campinas, SP, Brasil; 3 Seção de Hepatologia, Instituto Evandro Chagas - IEC, Secretaria de Vigilância em Saúde, Ministério da Saúde, Belém, PA, Brasil; 4 Centro Nacional de Primatas - CENP, Secretaria de Vigilância em Saúde, Ministério da Saúde, Ananindeua, PA, Brasil; 5 Departamento de Gastroenterologia LIM-07, Instituto de Medicina Tropical - IMT, Universidade de São Paulo, São Paulo, SP, Brasil

**Keywords:** Cestoda, cysticercus, liver, rodent, Taenia, Cestoda, cisticerco, fígado, roedores, Taenia

## Abstract

The study describes the occurrence of cysticercosis in liver of 22 wild agoutis (*Dasyprocta leporina*) in the Brazilian Amazon. The phylogenetic analysis and microscopic characteristics of metacestodes in liver tissue sections, associated with the geographic distribution of the intermediate hosts indicated that a possibly novel *Taenia* sp. metacestode caused the parasitism. Additionally, two cases of hepatic co-infection by *Taenia* sp., *Calodium* sp. and *Echinococcus oligarthra* were also observed among the analyzed animals. The results point to the need for a better understanding of hepatotropic parasites among wild rodents in the Brazilian Amazon.

## Resumo

O estudo descreve a ocorrência de cisticercose no fígado de 22 cutias (*Dasyprocta leporina*) silvestres da Amazônia brasileira. A análise filogenética e as características microscópicas dos metacestódeos em cortes histológicos de fígado, associadas à distribuição geográfica do hospedeiro intermediário, indicaram que, possivelmente, uma nova espécie de *Taenia* sp. Causou o parasitismo. Adicionalmente, dois casos de co-infecção por *Taenia* sp., *Calodium* sp. e *Echinococcus oligarthra* também foram observados entre os animais avaliados. Os resultados apontam para a necessidade de um melhor entendimento dos parasitas hepatotrópicos entre roedores selvagens da Amazônia brasileira.

The genus *Taenia* (Cestoda: Taeniidae) includes approximately 50 cestode species that are globally important in human and veterinary medicine (Loos-Frank, 2000; Hoberg, 2002, 2006; Nakao et al., 2013). The heteroxenous life cycle of these parasites involves mammals as intermediate as well as definitive hosts (Loos-Frank, 2000; Hoberg, 2002, 2006; Nakao et al., 2013). Infection with the larval (metacestodes) and adult forms of the parasite are referred to as cysticercosis and taeniasis, respectively (Hoberg, 2006; Nakao et al., 2013).

Taxonomic classification of *Taenia* spp. is frequently based on the morphological and morphometric characteristics of the parasites as well as the tissue distribution and the host species in which the cestodes are detected (Loos-Frank, 2000; Hoberg, 2002, 2006; Nakao et al., 2013). The morphology of the larval forms allows their classification into cysticercus, coenurus, strobilocercus, and fimbriocercus; however, this may not be sufficient for exact species identification in tissue sections (Hoberg, 2002; Chervy, 2002; Gardiner & Poynton, 2006; Eberhard, 2014).

Molecular analysis has, to date, supported the taxonomic classification of the members of family Taeniidae (Nakao et al., 2013). Investigating the phylogenetic relationships among taeniids has improved our overall understanding of epidemiology and more specifically the geographic distribution and life cycle of these parasites (Nakao et al., 2013).

In South America, pacas (*Agouti paca*) and agoutis (*Dasyprocta leporina*) are the main natural intermediate hosts for *Echinococcus vogeli* and *E. oligarthra* (syn. *E. oligarthrus*) (Vuitton et al., 2020; Souza et al., 2022). In humans, infection with these metacestodes causes the condition known as Neotropical echinococcosis (NE) (syn. Polycystic echinococcosis) (Vuitton et al., 2020; Souza et al., 2022).

**Figure 1:**
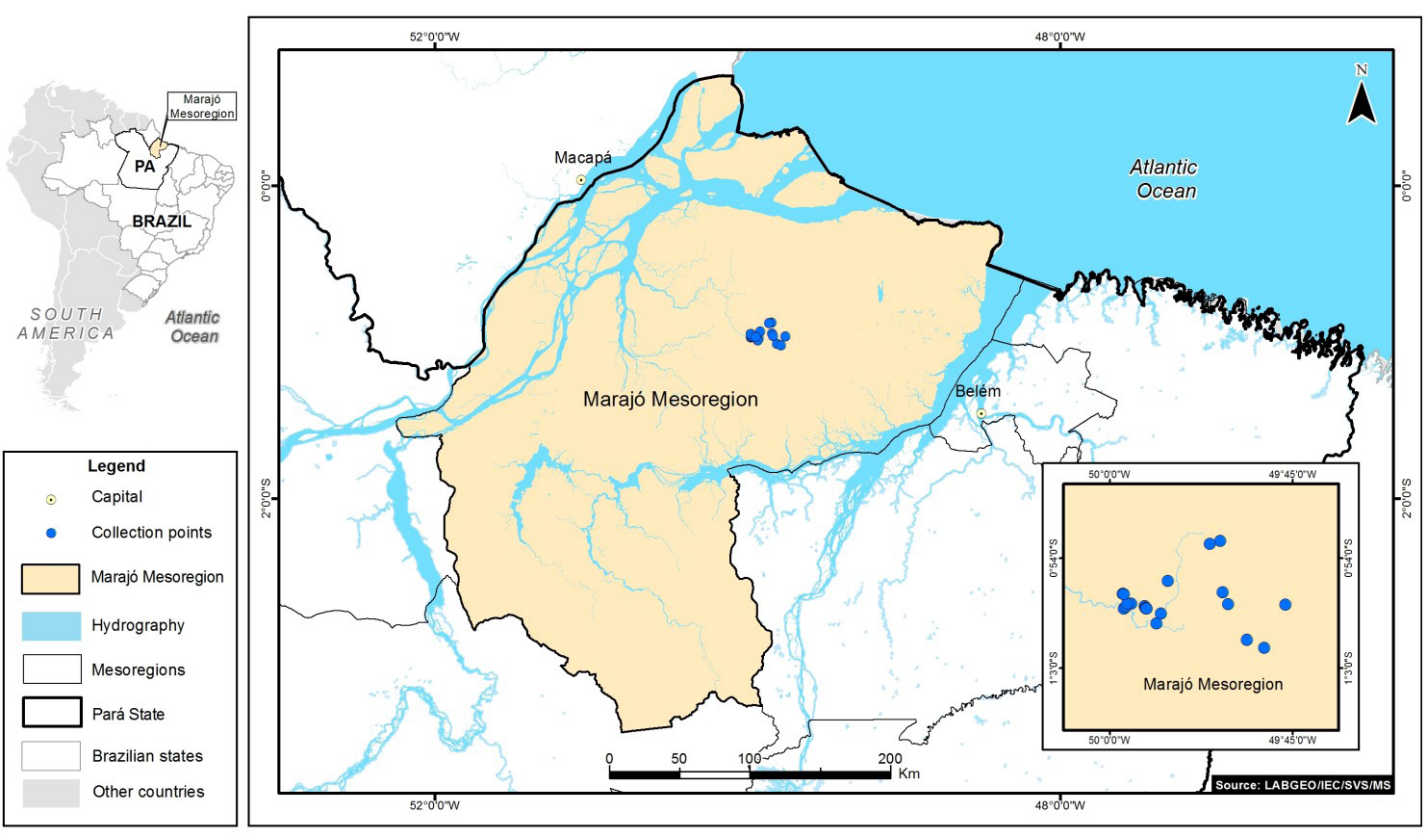
Sampling sites (blue spots) of 20 agoutis (*Dasyprocta leporina*) with hepatic cysticercosis on Marajó island, Brazilian Amazon, 2006-2009.

This study aimed to describe the occurrence of cysticercosis among free-ranging agoutis in the Eastern Brazilian Amazon. Tissue samples from 22 wild agoutis (*n* = 22) had initially been collected for another study. The fieldwork had been conducted between January 2006 and December 2009 in Anajás City on Marajó Island (Marajó mesoregion) in the Eastern Brazilian Amazon. The sampling sites for 20 of the 22 agoutis are shown in Figure 1. The aim of the initial study for which the samples had been obtained was to evaluate pacas and agoutis, the main intermediate hosts of NE, which were captured and slaughtered by native hunters for subsistence consumption in Anajás City. The viscera and carcasses of the animals had been inspected and samples had been collected.

On gross examination, in addition to polycystic lesions of *Echinococcus* spp. metacestodes (data not shown), the 22 agoutis exhibited lesions suggestive of cysticercosis. Single or multifocal cysts were discovered on the surface of the liver parenchyma, each no larger than 0.5 cm in diameter, with a translucid capsule containing a single white spot. These lesions were snap frozen in the field using liquid nitrogen (N_2_) and conserved at -70ºC for molecular analysis.

The cystic lesions were dissected, and DNA was extracted from the protoscolex of each using a QIAamp DNA Mini Kit (QIAGEN). The DNA obtained from the cysts in each animal was then subjected to PCR amplification of a 446-bp fragment of the COX-I gene (Bowles et al., 1992), and amplicons were detected by 1% agarose gel electrophoresis. All 22 samples yielded products that were close to the expected size. Diethylpyrocarbonate-treated water and *Echinococcus vogeli* DNA were included in the PCR assays as negative and positive controls, respectively.

The amplicons were sequenced (AB3500 Genetic Analyzer, Applied Biosystems) and Geneious v.8.1.3 software was used to extract consensus sequences from the DNA of the cysticerci. BLASTn analysis (Altschul et al., 1990) of these sequences revealed 85-91% nucleotide identity to other cestodes of the genus *Taenia* and 90-91% with *T. omissa*. Thus, based on the nucleotide sequences, the cysticerci from the agoutis were classified as *Taenia* sp. cysticercus and the sequences were deposited in GenBank under the accession numbers MG570191-570212.

**Figure 2:**
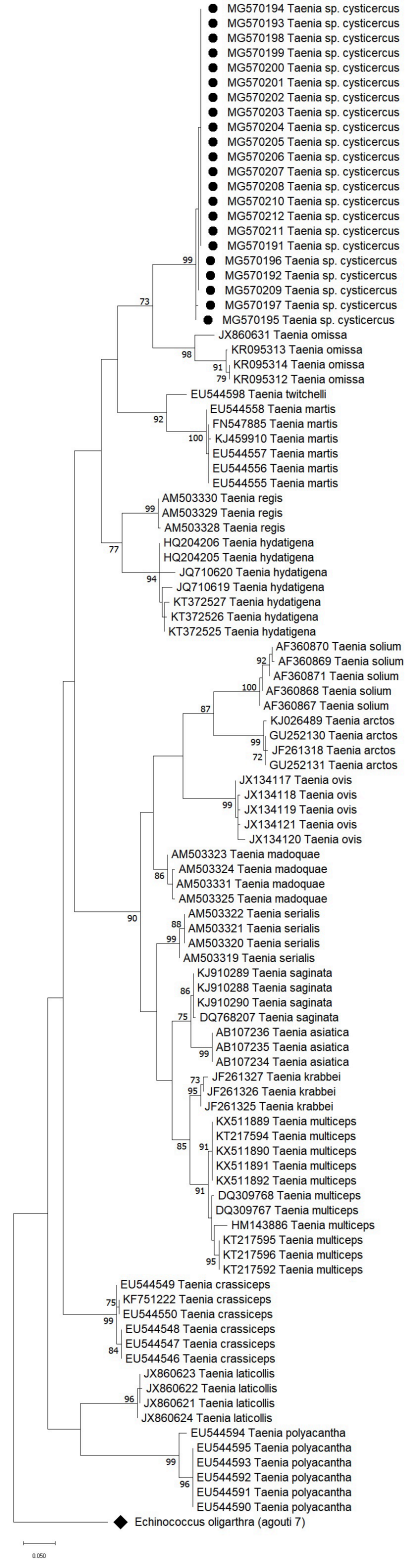
Maximum-likelihood (T92 + G) tree based on partial nucleotide sequences of Cox-I (328 bp) gene of 17 *Taenia* species. The sequences are identified by the Genbank accession number and the 22 agouti’s *Taenia* sp. cysticerci were highlighted (●). A sequence of *Echinococcus oligarthra* (♦) obtained in the study was used as an outgroup. Bootstrap values (1,000 replicates) > 70% are listed at the nodes.

The 22 nucleotide sequences were aligned with 82 representative sequences of the family Taeniidae, including 17 *Taenia* species, and a phylogenetic tree was constructed using the maximum likelihood method (T92 + G model, 1000 bootstrap replicates) with MEGA v.10.2.6 software. Phylogenetic analysis indicated that the sequences from the cysticerci in the 22 agoutis belonged to a single haplotype and formed a sister clade with sequences from *T. omissa* (Figure 2), suggesting that the cysticerci may belong to an as yet undescribed *T. omissa* variant or even a new *Taenia* species.

Histopathological examination under light microscopy (Eclipse Ni-U, Nikon) was also performed on liver tissue from the 10 agoutis that had exhibited multifocal cysticerci lesions. Liver tissue samples were collected, fixed in 10% formalin solution, and embedded in paraffin blocks that were then cut into 5-µm thick sections and stained with hematoxylin and eosin.

**Figure 3:**
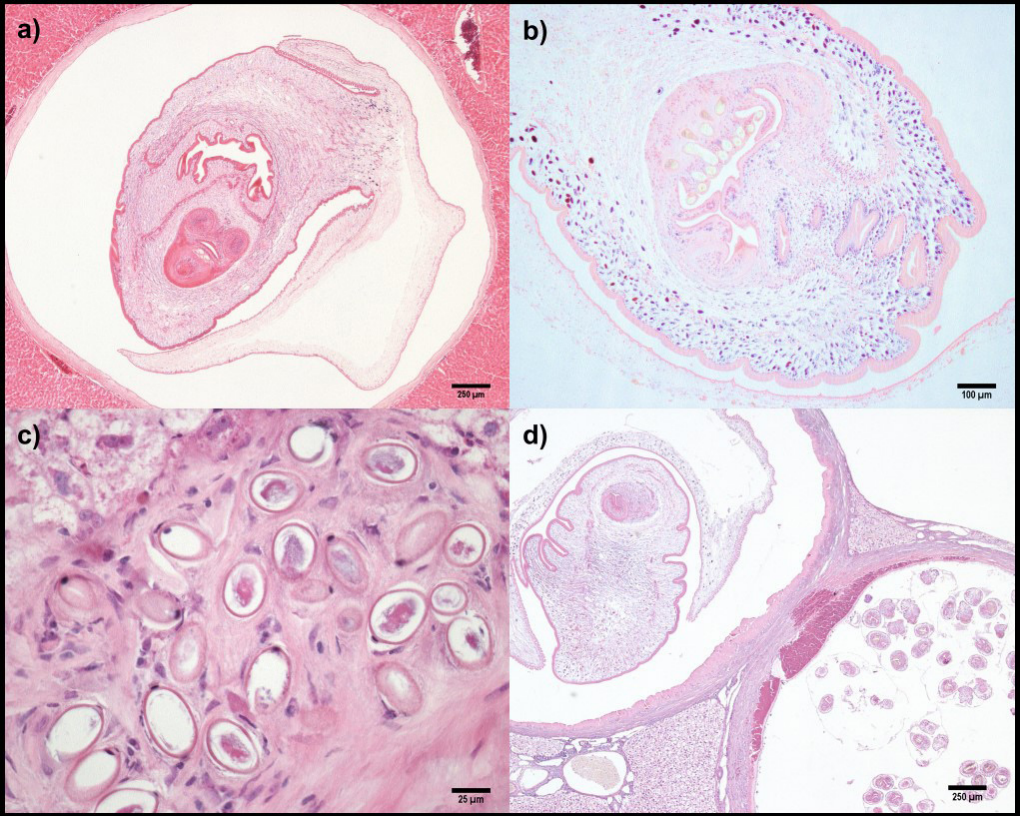
Microscopic liver sections of agoutis (*Dasyprocta leporina*) with cysticercosis. a) Cysticercus lesion containing a fluid-filled bladder and a single monocephalic metacestode. Case #4. Hematoxylin-eosin, 4x; b) Invaginated scolex presenting two rows of hooks on the rostellum. Developing suckers and multiple calcareous corpuscles are also observed. Case #13. Hematoxylin-eosin, 10x; c) Bioperculated ellipsoid-shaped eggs compatible with *Calodium* sp. surrounded by focal fibrosis. Case #7. Hematoxylin-eosin, 40x; d) Cysticercus lesion (upper left) containing a single *Taenia* sp. metacestode and a hydatid cyst (down right) containing multiple *Echinococcus oligarthra* protoscoleces. Case #7. Hematoxylin-eosin, 4x.

Microscopic examination revealed that the cystic lesions were composed of thick fibrous capsules that each contained a single metacestode (Figure 3a). The cestode larvae were found at different stages of development, but all showed characteristics common to monocephalic invaginated metacestodes, such as parenchymatous bodies containing calcareous corpuscles (Gardiner & Poynton, 2006; Eberhard, 2014). In tissue sections containing larvae at an advanced stage of development exhibited developing suckers, a bladder, and two rows of hooks in the invaginated scolex (Figure 3b), similar to other metacestodes (Gardiner & Poynton, 2006). Although measurement of the hooks was not feasible on histopathological sections, the metacestodes were morphologically compatible with cysticercus (Chervy, 2002; Gardiner & Poynton, 2006; Eberhard, 2014).

Taeniasis and cysticercosis occur commonly worldwide due to the number and variety of *Taenia* species and hosts (Loos-Frank, 2000; Hoberg, 2002; Eberhard, 2014). However, to the best of our knowledge, this is the first description of cysticercosis in agoutis.

The site of development of *Taenia* metacestodes seems to be species-specific (Hoberg et al., 2000; Eberhard, 2014), and cysticercosis lesions were observed only in the livers of the agoutis. Hepatic cysticercosis is rare in humans (Chaudhary et al., 2014), but in the natural intermediate hosts of several *Taenia* species (*T*. *hydatigena*, *T*. *pisiformis*, *T. regis*, *T*. *saginata*, *T*. *asiatica*, *T*. *saigoni*, *T*. *rileyi*), *Versteria mustelae*, and *Hydatigera taeniaeformis*, metacestodes may be detected in the liver and other tissues such as the lungs, skeletal muscles, mesentery, and abdominal cavity (Loos-Frank, 2000; Gardiner & Poynton, 2006; Nakao et al., 2013; Eberhard, 2014). Thus, agouti cysticerci may be hepatotropic, though additional studies are necessary to test this hypothesis.

In cattle, cysticercotic lesions range from viable, with absent or minimal inflammatory response, to degenerating, accompanied by the severe granulomatous response, necrosis, and calcification associated with the progressive destruction of metacestodes (Panziera et al., 2017). Although *Taenia* sp. metacestodes were observed at different stages of development, neither severe inflammatory reactions nor inviable metacestodes were detected. In 9 of the 10 cases, only a mild inflammatory infiltrate comprising eosinophils and few histiocytes and lymphocytes was observed surrounding the cysticerci and/or in the inner capsule of the cysts. Additional studies related to host-parasite interaction and tissue response will be needed to determine the length of time over which cysticerci can remain viable in the liver tissue of agoutis.

In addition to cysticercosis, histopathological evaluation of the 10 agoutis revealed co-infection with other hepatotropic parasites. The liver parenchyma of seven of the agoutis contained multifocal hyaline material deposits with elliptical, bioperculate eggs (Figure 3c), consistent with parasitism by *Calodium* sp. (syn. *Capillaria* sp.) (Gardiner & Poynton, 2006; Almeida et al., 2013; Eberhard, 2014; Delaney et al., 2018). Rodents are the main reservoirs of *Calodium* spp., and hepatic capillariasis has been described in a broad range of hosts worldwide (Delaney et al., 2018), including neotropical wild rodent species such as pacas and agoutis (Almeida et al., 2013; Jones et al., 2019).

Two agoutis showed evidence of infection by three species of parasites: *Taenia* sp., *Calodium* sp., and *Echinococcus oligarthra* (Figure 3d). Microscopically, the hepatic lesions of *Echinococcus* spp. metacestodes were characterized by the presence of hydatid cysts with internal germinal epithelium and multiple proligerous capsules containing protoscoleces (Gardiner & Poynton, 2006; Eberhard, 2014; Vuitton et al., 2020). Taxonomic identification of *E*. *oligarthra* in the liver tissues of both cases was performed using the same molecular protocol used for the cysticerci characterization (data not shown). Although co-infection with *Calodium hepaticum* and *Echinococcus vogeli* has been previously described in paca (Almeida et al., 2013), to the best of our knowledge, this is the first report of co-infection with *Taenia* sp., *Calodium* sp., and *E. oligarthra* in an agouti.

The co-infection by the metacestodes of *Taenia* sp. and *E. oligarthra* suggests that these parasites may share the same definitive host(s) on Marajó Island. Agoutis are prey for neotropical wild felids, such as ocelots (*Leopardus pardalis*) (Moreno et al., 2006; Emsens et al., 2014) and pumas (*Puma concolor*) (Moreno et al., 2006), and a previous survey of mammalian fauna reported the presence of ocelots, pumas, and jaguars (*Panthera onca*) in the area of Anajás City (Marques-Aguiar et al., 2002).

Infection with *T. omissa* was recently described in *P*. *concolor* in Brazil (Benatti et al., 2021). As these wild felids prey on agoutis, which are the intermediate hosts for *E. oligarthra* (Moreno et al., 2006; Vuitton et al., 2020), wild felid species may be the definitive hosts of *Taenia* sp. However, exposure of agoutis to the feces of other animals must also be considered.

*T. talicei* is considered an enzootic *Taenia* species in wild rodents from South America (Rossin et al., 2010). The phylogenetic relationship between the larval forms of *Taenia* sp. detected in agoutis and *T*. *talicei* could not be evaluated due to the absence of available nucleotide sequences from *T*. *talicei*, but these two parasites likely belong to different species because their larval forms have different hosts, unequal tissue distributions, and different morphological characteristics: *T*. *talicei* metacestodes are polymorphic, may present as fimbriocercus and polycephalic forms, and are found by the abdominal cavity of *Ctenomys* spp. rodents (Rossin et al., 2010), while the *Taenia* sp. cysticerci in the agoutis were monocephalic with a single armed invaginated scolex and were only observed in the liver of *D. leporina*.

Based on preliminary morphological and molecular analysis, geographic distribution, host characteristics, and tissue tropism, we hypothesize that the cysticercosis detected in agoutis on Marajó Island may be due to a new hepatotropic variant of *T*. *omissa* or even a novel *Taenia* species. Further parasitological and molecular studies are needed to evaluate the life cycle and to specify the geographic and host distribution of this *Taenia* sp., with the aim of clarifying species taxonomy and determining whether this taeniid represents another enzootic *Taenia* species in South America.

## References

[B001] Almeida F, Caldas R, Corrêa C, Rodrigues-Silva R, Siqueira N, Machado-Silva JR (2013). Co-infections of the cestode *Echinococcus vogeli* and the nematode *Calodium hepaticum* in the hystricomorphic rodent *Agouti paca* from a forest reserve in Acre, Brazil. J Helminthol.

[B002] Altschul SF, Gish W, Miller W, Myers EW, Lipman DJ (1990). Basic local alignment search tool. J Mol Biol.

[B003] Benatti D, De Santi M, Werther K, Tebaldi JH, Hoppe EGL (2021). Helminthfauna of road-killed cougars (*Puma concolor*) from the Northeastern region of São Paulo State, Brazil. Braz J Vet Parasitol.

[B004] Bowles J, Blair D, McManus DP (1992). Genetic variants within the genus *Echinococcus* identified by mitochondrial DNA sequencing. Mol Biochem Parasitol.

[B005] Chaudhary V, Bano S, Kumar P, Narula MK, Anand R (2014). Hepatic cysticercosis: a rare entity. Abdom Imaging.

[B006] Chervy L (2002). The terminology of larval cestodes or metacestodes. Syst Parasitol.

[B007] Delaney MA, Treuting PM, Rothenburger JL, Terio KA, McAloose D, Leger JS (2018). Pathology of wildlife and zoo animals.

[B008] Eberhard ML, Bowman D (2014). Georgis’ parasitology for veterinarians.

[B009] Emsens WJ, Hirsch BT, Kays R, Jansen PA (2014). Prey refuges as predator hotspots: ocelot (*Leopardus pardalis*) attraction to agouti (*Dasyprocta punctata*) dens. Acta Theriol.

[B010] Gardiner CH, Poynton SL (2006). An atlas of metazoan parasites in tissue section.

[B011] Hoberg EP, Jones A, Rausch RL, Eom KS, Gardner SL (2000). A phylogenetic hypothesis for species of the genus *Taenia* (Eucestoda: taeniidae). J Parasitol.

[B012] Hoberg EP (2006). Phylogeny of *Taenia*: species definitions and origins of human parasites. Parasitol Int.

[B013] Hoberg EP (2002). *Taenia* tapeworms: their biology, evolution and socioeconomic significance. Microbes Infect.

[B014] Jones KR, Lall KR, Garcia GW (2019). Endoparasites of selected native non-domesticated mammals in the Neotropics (New World Tropics). Vet Sci.

[B015] Loos-Frank B (2000). An up-date of Verster’s (1969) Taxonomic revision of the genus *Taenia* Linnaeus’ (Cestoda) in table format. Syst Parasitol.

[B016] Marques-Aguiar SA, Melo CCS, Aguiar GFS, Queiroz JAL (2002). Preliminary survey of the mammalian fauna in the Anajás-Muaná region, Marajó Island, Pará State, Brazil. Rev Bras Zool.

[B017] Moreno RS, Kays RW, Samudio R (2006). Competitive release in diets of ocelot (*Leopardus pardalis*) and puma (*Puma concolor*) after jaguar (*Panthera onca*) decline. J Mammal.

[B018] Nakao M, Lavikainen A, Iwaki T, Haukisalmi V, Konyaev S, Oku Y (2013). Molecular phylogeny of the genus *Taenia* (Cestoda: Taeniidae): proposals for the resurrection of *Hydatigera* Lamarck, 1816 and the creation of a new genus *Versteria.*. Int J Parasitol.

[B019] Panziera W, Vielmo A, Bianchi RM, Andrade CPD, Pavarini SP, Sonne L (2017). Aspectos macroscópicos e histológicos da cisticercose bovina. Pesq Vet Bras.

[B020] Rossin MA, Timi JT, Hoberg EP (2010). An endemic *Taenia* from South America: validation of *T. talicei* Dollfus, 1960 (Cestoda: Taeniidae) with characterization of metacestodes and adults. Zootaxa.

[B021] Souza LS, Sampaio R, Gomes APN, Morato RG, Chiarello AG, Souza LS (2022). Occurrence of potential wild hosts of *Echinococcus vogeli* in the forests of southwestern Brazilian Amazonia. Biota Neotrop.

[B022] Vuitton DA, McManus DP, Rogan MT, Romig T, Gottstein B, Naidich A (2020). International consensus on terminology to be used in the field of echinococcoses. Parasite.

